# Health system resilience during COVID-19 understanding SRH service adaptation in North Kivu

**DOI:** 10.1186/s12978-022-01443-5

**Published:** 2022-06-06

**Authors:** Lara S. Ho, Maria Paola Bertone, Wesam Mansour, Cyprien Masaka, Jessica Kakesa

**Affiliations:** 1 International Rescue Committee, Health Unit and ReBUILD for Resilience, Washington, DC USA; 2 Institute for Global Health and Development, Queen Margaret University and ReBUILD for Resilience, Edinburgh, UK; 3 Department of International Public Health and ReBUILD for Resilience, Liverpool School of Tropical Medicine, Pembroke Place, Liverpool, L3 5QA UK; 4 International Rescue Committee, Goma, Democratic Republic of Congo

**Keywords:** COVID-19, Sexual and reproductive health, Democratic Republic of the Congo, Adaptation

## Abstract

**Background:**

There is often collateral damage to health systems during epidemics, affecting women and girls the most, with reduced access to non-outbreak related services, particularly in humanitarian settings. This rapid case study examines sexual and reproductive health (SRH) services in the Democratic Republic of the Congo when the COVID-19 hit, towards the end of an Ebola Virus Disease (EVD) outbreak, and in a context of protracted insecurity.

**Methods:**

This study draws on quantitative analysis of routine data from four health zones, a document review of policies and protocols, and 13 key-informant interviews with staff from the Ministry of Public Health, United Nations agencies, international and national non-governmental organizations, and civil society organizations.

**Results:**

Utilization of SRH services decreased initially but recovered by August 2020. Significant fluctuations remained across areas, due to the end of free care once Ebola funding ceased, insecurity, number of COVID-19 cases, and funding levels. The response to COVID-19 was top-down, focused on infection and prevention control measures, with a lack of funding, technical expertise and overall momentum that characterized the EVD response. Communities and civil society did not play an active role for the planning of the COVID-19 response. While health zone and facility staff showed resilience, developing adaptations to maintain SRH provision, these adaptations were short-lived and inconsistent without external support and funding.

**Conclusion:**

The EVD outbreak was an opportunity for health system strengthening that was not sustained during COVID-19. This had consequences for access to SRH services, with limited-resources available and deprioritization of SRH.

**Supplementary Information:**

The online version contains supplementary material available at 10.1186/s12978-022-01443-5.

## Plain Language Summary

Women and girls often face increased challenges to accessing healthcare during epidemics on top of pre-existing health disparities. There is emerging evidence that COVID-19 has had negative impacts on the health of women and girls in sub-Saharan Africa due to diverted funding, reduced services, negative socioeconomic impacts, and increased or new barriers to access. In the DRC, COVID-19 hit shortly after the end of an Ebola epidemic within a context of protracted insecurity. This study used mixed methods and drew upon 13 interviews to examine the effects of COVID-19 on SRH services in North Kivu and how the health system did or did not adapt to ensure continued access and utilization of SRH services. There was limited prioritization of SRH during COVID-19. Although the government issued policies on how to adapt SRH services, these were developed centrally, without much guidance on how to operationalize these policies in different contexts. Consequently, healthcare providers and civil society actors developed their own ways to continue activities at local levels, not necessarily in a systematic way. There was limited longer-term strengthening of the health system that could adapt to the subsequent COVID-19 pandemic aside from increased capacity of healthcare providers to manage infection prevention and control measures. However, this was hampered by the lack of personal protective equipment that received no external support. Therefore, donors need to consider how resources can be leveraged to support sustained strengthening of the health system to be able to adapt to shocks even when resources are limited.

**Supplementary Information:**

The online version contains supplementary material available at 10.1186/s12978-022-01443-5.

## Background

There is often collateral damage to health and health systems during epidemics, and women and girls frequently bear the burden with reduced access to non-outbreak related services. In humanitarian settings, where health systems struggle to deliver health services to populations already affected by violence, natural disaster, or political instability, the emergence of COVID-19 further threatens the delivery, quality, accessibility, and availability of vital care to those communities. In those settings, access and use of sexual and reproductive health (SRH) services among women and girls is limited, even when services are available [1]. COVID-19 has exacerbated this poor access and had disastrous consequences for many in the global South [2].

Past humanitarian crises have shown that reduced access to family planning, abortion, antenatal care (ANC), human immune-deficiency virus (HIV), sexual and gender-based violence (SGBV), and mental healthcare services results in increased rates and sequelae from unintended pregnancies, unsafe abortions, sexually transmitted infections (STIs), pregnancy complications, miscarriage, post-traumatic stress disorder, depression, suicide, intimate partner violence, and maternal and infant mortality [3, 4]. During the 2014 Ebola Virus Disease (EVD) outbreak in Africa, SRH programming was given limited attention in the outbreak response, leading to sharp declines in service utilization. Consequently, excess maternal and neonatal deaths exceeded the number of deaths from Ebola [5, 6].

In the Democratic Republic of the Congo (DRC), the prevalence of the modern contraceptive is only 8%, and the unmet need is 28% among women aged 15–49 years [7]. Abortion rates in DRC is 56 per 1,000 women aged 15–49 in comparison to the estimated global average of 35 abortions per 1,000, which are probably unsafe abortions, given the social-normative culture in the DRC. Moreover, with the poor post-abortion care utilisation is the country, complications from unsafe abortions may contribute to the high maternal mortality rate, reached to 846 maternal deaths per 100,000 live births [8]. According to the Demographic and Health Survey in 2014, 18% of adolescents aged 15–19 had begun childbearing in North Kivu, with Stigma related to pregnancy outside of marriage is high in the province. An assessment conducted in Masisi, North Kivu in 2013 showed that out of the 26 assessed public health facilities, none of them had a trained health worker to provide SRH services to adolescent girls [9].

With the recognition of reproductive health as a human right, the Inter-Agency Working Group (IAWG) on Reproductive Health drafted a list of minimum reproductive health interventions that would be put in place in the event of emergency. These interventions are now packaged as the Minimum Initial Services Package (MISP) and are targeted to prevent excess morbidity and mortality among women and girls [10]. The MISP objectives include prevention of HIV transmission, reducing the morbidity and mortality due to HIV and other STIs, planning for comprehensive SRH services and their accessibility, and the prevention and management of SGBV [10]. Evaluations of SRH in emergencies, especially since the development of the MISP, have demonstrated a greater awareness of the critical role of SRH services and their evaluations by humanitarian organizations, Ministries of Health (MOH), and field responders [11].

The COVID-19 pandemic has required SRH practitioners also to be adaptive and innovative to meet population needs, particularly those of women and girls [12]. Moreover, adaptive leadership has also been necessary during the COVID-19 pandemic as policymakers, practitioners, and researchers work to provide key SRH services to populations in humanitarian and fragile settings in a way that understands the needs and realities of communities and their contexts. In their guidance for adapting interventions to meet gender needs during the COVID-19 outbreak, Ramalingam et al. assert the need to identify which interventions, or combinations of such, are most effective and will have the best intended impact and how to ensure these interventions can be designed to adapt to change. They also demonstrate that policymakers need to rapidly interpret different forms of evidence, determine exactly what they seek to achieve, define measures that will trigger adaptations to interventions, and most importantly bring communities into the decision-making process [13].

In North Kivu province of the DRC, the COVID-19 pandemic hit in March 2020, with increasing number of cases and subsequent (total or partial) lockdowns and restrictions in April and May–August. The pandemic hit towards the end of an Ebola outbreak (August 2018–June 2020) in a context of protracted insecurity due to decades of conflict between armed non-state actors and the government. The country remained ill-equipped to address COVID-19 as there was a shortage of facilities to deal with the pandemic. Of 26 provinces, COVID-19 testing was only available in the towns of Kinshasa, Matadi, Lubumbashi, Goma, Kolwezi, and Mbandaka. The daily testing of only 900 individuals was very low compared with a population of more than 100 million [14]. While research is emerging on the impact of COVID-19 in other settings, there has been limited analysis of the humanitarian context in eastern DRC. Little evidence is also available about health service delivery in such challenging context and how SRH services are delivered for girls and women [15]. In North Kivu, challenges contributed to poor SRH outcomes also include the conservative culture that imposed some gender norms and patriarchal control over girls and women which contributed to poor SRH outcomes [8]. In addition, few analyses so far have used a policy analysis approach looking at the policy making processes around SRH response and adaptations.

Therefore, this study aimed to understand the impact of the pandemic on access to SRH services, how SRH protocols and practices were or were not adapted to respond to women and girls’ needs during the COVID-19 pandemic, and to identify the challenges to effective SRH service adaptation in North Kivu.

## Methods

### Study design and data sources

A mixed-methods approach that used a largely retrospective (rapid) case study design was adopted to gather qualitative and quantitative secondary information as well as the views of stakeholders about how SRH policies, protocols and practices were adapted in response to COVID-19. Data sources included a document review, key-informant interviews, quantitative secondary data analysis, and knowledge embedded within the research team.

### Data collection

Documents included in the document review were found through a purposeful online search (generally and specifically using the database of the *Cellule d’Analyse en Sciences Sociales* (CASS) [16], as well as shared by International Rescue Committee (IRC) staff in DRC and key-informants. The document review included 17 documents and included existing laws, policies, and protocols for SRH in DRC and North Kivu (n = 4); descriptions of COVID-19 policy response and adaptations (n = 4); presentations from Health Cluster meetings (n = 1); other reports and analyses of the COVID-19 effects on SRH and on the health system in DRC (n = 2); and reports on SRH and on the health system of North Kivu before COVID-19 (n = 6). In addition, quantitative data from four health zones (Goma, Karisimbi, Beni, and Mweso) in North Kivu were extracted from DRC’s District Health Information Software-2 (DHIS2) database for the period between 2019 and 2020 and shared with the study team. The indicators reviewed were maternal deaths, antenatal care visits, skilled birth attendance, and new consultations.

Twenty-three key informants were purposefully identified from the five following groups: representatives of United Nations (UN) agencies, Ministry of Public Health (MSP), international non-governmental organizations (INGOs), national non-governmental organizations (NNGOs), and civil society organizations (CSOs). The overall number of respondents captured covered a high proportion of key actors and these five groups were selected to represent the main stakeholders involved in delivery of health services in the province, with a target of five organizations from each group, with the exception of the UN, which only had three relevant agencies to include. The respondents approached for interviews were those responsible for reproductive health within their organizations. A list of the representatives of main actor’s organizations in each of these categories was initially prepared by IRC staff in Goma with the aim of five per category. A total of 23 key informants were selected and contacted. Interviews were successfully scheduled with 13 of those key informants, of whom only one was female (Table 1).

**Table 1:** Key informants contacted and interviewed

Category	Target number	Number interviewed
UN	3	2
MSP	5	3
INGOs	5	2
NNGOs	5	4
Local CSOs	5	2
Total	23	13

An information sheet and consent form in French were provided to the respondents at the time of scheduling interviews with all information needed for the interview. The informed consent was collected for all the interviewees including consent to recording of interviews or taking notes. Investigators conducted interviews with key-informants in French—two of the interviewers do not have working relationship with the respondents (LSH and MPB), while one (CM) does. Additionally, the research team included investigators from both academic institutions (MPB, WM) and an implementing agency (NGO) (LSH, CM, JK), and from both the outside (LSH, MBP, WM) and inside the country (CM, JK). The mix of insiders and outsiders with iterative discussions between researchers at data collection and analysis stages, including reflections on researchers’ positionality, were chosen to strengthen the rigor and trustworthiness of our qualitative findings [17] by allowing for both external and internal perspectives on the context. Each interview lasted about 1-h and was done either remotely using Whatsapp or Microsoft Teams or in person, between December 2020 and January 2021 guided by a semi-structured interview guide which focused on understanding the response to COVID-19 in terms of changes to SRH services, actors responsible for those changes, as well as on unpacking the decision processes on changes and their implementation at different levels of the health system and their impact (Additional file 1: Annex 1). While interviews were not transcribed or translated, detailed notes were taken given the rapid approach of the case study. Illustrative quotations used in the results section below were transcribed and translated by the authors based on the audio recordings.

### Data analysis

Quantitative secondary data were reviewed for trends between 2019 and 2020 using Microsoft Excel to address the question on the impact of COVID-19 on utilization of SRH services and on SRH outcomes for women and girls. Data was also triangulated with reports and other information to compare trends in North Kivu with those at the national level and interpret those trends against the COVID-19 epidemic data and other key factors, such as changes in funding levels.

Interviews and documents were analyzed using thematic analysis [18, 19]. A series of themes/codes were developed building on the aims of the study but allowing space for revisions during analysis to accommodate emerging themes (Additional file 1: Annex 2). Codes were applied to interviews’ field notes and documents using an Excel-based extraction matrix. Findings from each source were carefully integrated and triangulated, and results of the data analysis, document review and interviews were analyzed and written up jointly to allow for complementarity between data sources. The preliminary results of the study were discussed between researchers as well as shared with participants during a Health Cluster SRH sub-working group meeting in Goma for their feedback. Furthermore, the final report was sent to participants.

## Results

### Impact of COVID-19 on access to SRH services and needs

Respondents reported a decrease in health services’ utilization at the beginning of the pandemic in March 2020. There were a variety of explanations as to why: the risk of being identified as having COVID-19 and subsequently forced into an isolation center making people fearful of seeking care; lockdown rules made it difficult for people to travel to facilities; limits on the number of people allowed to gather at facilities causing increased waiting times; some SRH services being regarded as non-essential and therefore access to facilities refused; reduction in sensitization activities because of restrictions on gatherings; and economic constraints to paying user fees, as financial hardship increased during lockdowns that reduced movements and constrained economic activities.

Utilization rates seemed to recover by August 2020 as lockdowns eased and community sensitization lessened fears of being quarantined or infected; people became used to the measures taken to reduce risk of infection such as checkpoints, mask requirements, and reduced size of gatherings; and providers adapted services. One respondent noted that community members saw COVID-19 as being imported unlike EVD, and because it was not as fatal and visible, it was harder to sensitize the community to the dangers. Another stated:

*“When COVID started, people were scared of everything. Because in our province, Ebola, it had been here more than a year. So, people knew Ebola and had become accustomed to living with Ebola. So, when COVID arrived, we presented it as a disease that you could get from someone when they breathed or spoke. So, this may have been why utilization decreased...” (Male_UN agency)*

**Fig. 1:**
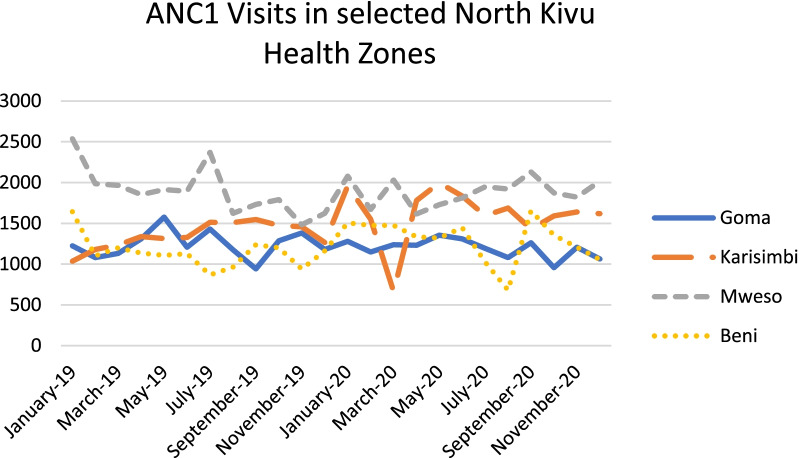
ANC1 Visits in selected North Kivu Health Zones

**Fig. 2:**
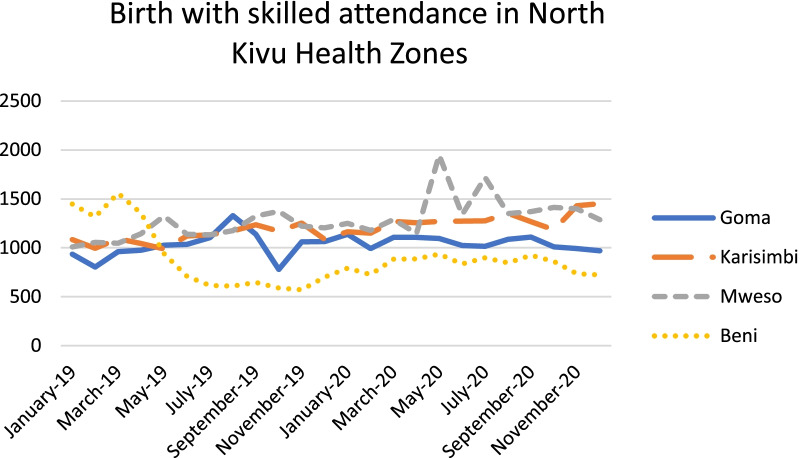
Birth with skilled attendance in North Kivu Health Zones

**Fig. 3:**
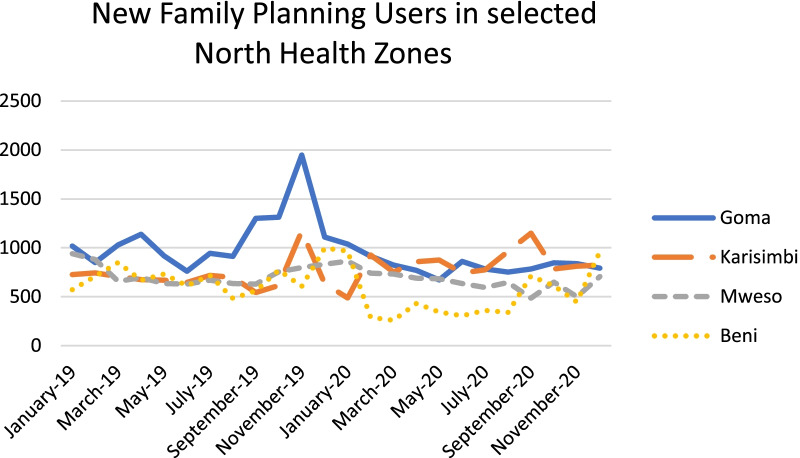
New Family Planning Users in selected North Health Zones

Quantitative utilization data from four health zones reflect the qualitative findings for the most part with some initial decreases around April 2020 that recovered later in the year. Factors such as the end of free health services when Ebola funding ceased at the end of July 2020, and insecurity in some health zones may have caused some of the other fluctuations observed in Figs. 1, 2, and 3. At least one NGO respondent noted that the effects varied in relation to how a particular health zone was affected by COVID-19; in health zones where there were no reported cases many people did not believe in the seriousness of the illness so it may not have had much impact on patient behavior.

Respondents felt that populations without safety nets, such as internally displaced and street children were particularly at risk because of loss of livelihoods. User fees were not reduced or removed as they had been during the earlier EVD epidemic.

Respondents reported increases in women seeking family planning and abortion care later in the pandemic, which they attributed to women deciding that it was not financially prudent to be having more children, and to increases in unintended pregnancies resulting from increased transactional sex for survival and sexual activity among adolescents because of school closures. This also led to increased STIs according to some respondents.

### COVID-19 policy response and guidelines

As the reach of the pandemic became clear around the world and in the DRC, the government put together guidance to plan a COVID-19 [20] response including guidance on SRH services [21], focused mostly on infection prevention and control (IPC) measures to reduce infections of health workers and patients, and on IPC-related adaptations to providers’ behaviors in clinical settings, as well as remote consultations and promoting self-care. However, no information is provided on procedures to ensure these adaptations are implemented in practice. In addition, in contrast to what had happened with the EVD epidemic, there was no official policy to reduce user fees, which in the DRC are charged to patients for almost all services with few exceptions. In the context of the lockdown and the loss of livelihoods for many, continuing to charge user fees would have created barriers to access services for many girls and women.

During interviews, there was a sense that decision-making happened in a top-down manner. Respondents stressed that it was the government and the MSP at the national level in charge of decision-making and drafting policies and guidelines, which were then communicated to the provinces and from there to the zonal and facility levels. It appears that in North Kivu, the provincial health authority (DPS) remained almost a “paper pusher”, tasked with sharing the documentation and guidelines from the government at the national level to the partners in charge of implementation, but without real ownership of the response and dependent on partners for financial support to facilities. Communities and civil society, whose role had been stressed and built on during the response to the EVD epidemic, did not seem to play an active and critical role for the planning of the COVID-19 response [22].

Respondents also noted a lack of funding, technical expertise, and overall momentum among stakeholders, especially when compared to the EVD response, which created challenges for effective coordination and policies implementation. One respondent said:

*“The effort of the decision makers was not there after putting in place policy” (Male_CSO)*

Another challenge mentioned was the lack of prioritization of SRH during COVID-19, for example as funds at the provincial level originally earmarked for family planning were diverted to the COVID-19 response.

### Adaptations to SRH services in practice and challenges in the response

Despite the lack of coordinated support from central and provincial levels, some ad-hoc pragmatic adaptations to SRH services at zone, facility, and community level were mentioned in interviews. These included providing several months of oral contraceptives so that women would not have to come back as often to replenish their supply, increasing the number of community sensitization sessions, scheduling appointments by phone to avoid waiting times, and extending health facility hours to reduce crowding. In some facilities, ANC and immunization sessions were increased to several days a week to reduce the people’s number per session in line with public health restrictions.

While adaptations appeared to be generally accepted and pragmatic, respondents reported that some women did not like the increased wait times that resulted, and that some pregnant women felt that wearing masks was very uncomfortable. The need to increase the number of sessions for ANC and other group services or to extend hours was also a strain on providers who had to work longer hours.

However, adaptations to SRH services remained mostly ad hoc and were not uniformly implemented. A main source of variation was the extend of the support available for training and supervision. One respondent said:

*“It’s a continuous process, when an organization (an NGO) comes to support a facility, they come with a package of support and with that package of course a protocol and they start explaining to us and with training, that with the package this is now the protocol, and then we start implementing according to these protocols” (Male_MSP)*

Another source of variation in SRH adaptations was the financial support to facilities or community-level organizations due to differences in funding partners. In addition to limiting training and supervision, the lack of funding impacted the availability of supplies for both the COVID-19 response and IPC, as well as to ensure SRH services could continue to be delivered for free. The lack of additional funding combined with increased workloads led to some demotivation of healthcare workers.

Additional challenges to the adaptation of SRH services came from the longstanding issues relating to conflict and instability in some areas.

A recurrent comparison that respondent made was between the EVD and the COVID epidemics. While it was mentioned that the experience of responding to EVD had prepared providers and the Health Cluster coordination for COVID-19 for example by establishing IPC measures and protocols and raising awareness of their importance among health workers, patients and clients, respondents noted a stark difference between the responses to the two epidemics, with EVD better funded, better coordinated and evidence-based, while COVID lacked funding and had top-down policies with fragmented implementation of measures. This left some of the respondents with a sense of a missed opportunity for health system strengthening.

*“The health system remains extremely fragile. In principle, the health system was supposed to take advantage of the Ebola epidemic for its strengthening. And indeed, there has been some capacity strengthening [during Ebola]. However, when the new COVID crisis arrived and people [NGOs/partners] left, we had the impression that the system is again fragile. So, it was a wrong impression that the system could have been strengthened. Quite the opposite!” (Male_KI-UN2)*

## Discussion

The access and use of SRH services among women and girls in LMICs and humanitarian settings is limited, even when services are available [1]. In Sub-Saharan Africa (SSA), access to SRH services is generally poor and the emergence of COVID-19 has further exacerbated this limited access [2]. The COVID-19 pandemic has resulted into redirection of funds and attention by governments, donors, and stakeholders towards COVID-19 containment efforts, thereby diverting focus from other important issues including SRH. This has led to women and girls being less able to access important healthcare services, while at the same time their SRH needs are likely to have increased [23], resulting in increased risk of maternal morbidity and mortality.

Similar to global findings, women and girls in North Kivu were reported to have decreased access to important healthcare services, whereas their SRH needs are likely to have increased [23]. Although respondents noted a decrease in health services’ utilization at the beginning of the pandemic, rates recovered by August 2020 after successful community sensitization and people becoming familiar with restrictions and safety measures. This is in line with the findings of the CASS report [24]. On the one hand, there was an increase in the use of family planning services by women after an initial reduction because of fear of becoming pregnant due to the cost of raising a child and an increase in sexual activity among adolescents and transactional sex during COVID-19; but reduced ANC visits which they did not always consider urgent, especially by women who had already given birth [24]. Transactional sex is a coping strategy commonly used by women and people with different gender identity to survive during extreme hardship, insecurity and economic instability e.g., during COVID-19. It was frequently reported during EVD-outbreak, however, it makes those vulnerable groups more prone to exploitation, HIV and SGBV [25, 26].

Many health programmes in humanitarian settings have made several SRH adaptations to respond to women and girls needs during COVID-19. An International Planned Parenthood Federation (IPPF) programme that is implemented across 15 countries in Africa and Asia recommended adapting clinic services in line with the national guidelines on social distancing and IPC measures [12]. In the DRC, the country had put together guidance to plan a COVID-19 response. Learning from the EVD-outbreak, the focus was on the IPC measures and its implementation in clinical settings to limit the infection, in addition to masks. However, this guidance was largely top-down, with limited detail on operationalization of how to ensure continued access to services and no concerted effort to support SRH services’ adaptations. Some measures that were implemented in other countries e.g., community distribution of family planning, increased support for self-care, and mHealth solutions do not seem to be used in this context [12]. This may be due to limited financial and human resources, poor technological infrastructure, and policy barriers to task-shifting. Communities and civil society, whose role had been stressed and built on during the EVD response, did not seem to play an active role for the planning of the COVID-19 response.

Nevertheless, as is often the case in the DRC, health zone and facility level staff developed their own adaptations to become more resilient [27] such as: setting specific appointments to patients to reduce the waiting time, using telephone communication, and reducing group counselling to 5 people maximum. These adaptations could overcome problems of rare clinic appointments and long waiting times for ANC, contraceptive counselling, or other SRH services, which increased risk of infection transmission [28]. Similarly, IPC measures were already known due to EVD prevention, however, diminishing support and funding to keep them in place, coupled with a perception of COVID-19 as less threatening compared to EVD, had led to the abandonment over time of many IPC practices. This also forced the health zones and organizations supporting them to come up with their own ad hoc strategies to adapt services to ensure continued access to SRH services. These included providing several months of oral contraceptives so that women would not have to come back as often to replenish their supply, increasing the number of sensitization sessions in the community to inform people of how to continue to seek services safely and to protect themselves from COVID-19, scheduling appointments by phone to avoid waiting times, and extending health facility hours to reduce crowding.

Despite the local level ingenuity, adaptations were not consistent and were short-lived in the absence of external support and funding. The stark difference in funds available was reported by most respondents, but there was also less coordination, technical support, capacity for innovation, learning culture, and multi-sectoral collaboration. The lack of external support for training and supervision meant that facilities were less likely to have implemented the new protocols and consistently followed guidance, particularly if they did not have external NGO partner support. The lack of funding has also impacted the availability of supplies both for COVID-19 response which resulted in stockouts, both for IPC/PPE materials as well as SRH commodities, and this in turn threatened the ability of SRH services to continue to be delivered for free. The logistical and technical support and the capacity for coordination and standardization of practices was also limited during the COVID-19 response compared to the EVD response. Likewise, in other Sub-Saharan Africa countries, accessing SRH services including ANC services, has been impacted in Kenya, Tanzania and Uganda with consistently high maternal and neonatal mortality rates due to limited healthcare resources, restrictions in movement, and a shortage of healthcare workers due to the COVID-19 response [23]. Shortage of funding has also limited the coordination and the overall momentum among agencies and stakeholders, and this ineffective coordination has led to non-standardized and patchy implementation of the COVID-19 guidelines in the facilities and communities they supported.

Several respondents expressed frustration that the unsystematic and weak response demonstrated how the EVD response had limited impact on strengthening the health system as it was still dependent on external funding and support. This reflects the continued gaps in support from the central government to provincial and zonal health teams and the continued challenges in reinforcing bottom up strengthening approaches in a context of limited resources all around, volatility of external support, and continued fragility.

However, on the positive side, the experience with EVD had prepared providers and the health cluster coordination for COVID-19. As described before, healthcare providers and communities demonstrated resilience in doing what they could to safely distance and continue services with the resources they had.

### Limitations of the study

This study had some limitations that need to be considered. Conducting data collection during travel restrictions presented a challenge to engaging patients in the empirical study. As most interviews were conducted remotely, we were only able to consult program implementation staff in designing the study, although it was informed by an earlier evaluation on the impact of Ebola on SRH services in North Kivu that included community members’ experiences [29]. It was also difficult to schedule many interviews due to end-of-year holidays and competing crises (new EVD-outbreak and attacks on civilians) therefore, we ended up only with 13 interviews. While these interviews happened during the pandemic, the questions posed asked respondents to reflect on past events, and there may have been biases in what they recalled. As is often the case with use of routine HMIS data, quality can always be a concern, although these were from facilities supported by an NGO that provided support and review of data quality.

## Conclusions

The EVD outbreak (a second one erupted during the course of data collection) was an opportunity for health system strengthening that in the end was not sustained through the COVID-19 pandemic. This had consequences for access to SRH services, as the limited resources available for responding to the pandemic resulted in SRH services’ deprioritization despite increased needs. This can have impacts on increased maternal morbidity and mortality. Respondents advised that continued funding was needed to support SRH services. Donors and future research should consider how resources can be leveraged to support sustained health system strengthening to be able to absorb shocks and prevent increased risk for mortality even when new influxes of funding during a crisis such as a new outbreak are limited.

## Supplementary Information

**Additional file 1.** **Annex 1:** Interview Guides in French and English. **Annex 2:** Coding Framework.

## Data Availability

The datasets used and/or analyzed during the current study are not publicly available due to confidentiality and ethical restrictions but are available from the corresponding author on reasonable request.

## Abbreviations

ANC: Antenatal care
CASS: Cellule d’Analyse en Sciences Sociales
CSO: Civil Society Organization
DRC: Democratic Republic of Congo
EVD: Ebola Virus Disease
HIV: Human immune-deficiency virus
IAWG: Inter-Agency Working Group
(I)NGO: (International) Non-Governmental Organization
IPC: Infection prevention and control
IPPF: International Planned Parenthood Federation
IRC: International Rescue Committee
LMIC: Low-to-Middle-Income Country
MISP: Minimum Initial Service Package for Reproductive Health in Emergencies
MNCH: Maternal, Newborn, and Child Health
MOH: Ministry of Health
MSP: Ministry of Public Health
PPE: Personal protective equipment
SGBV: Sexual and gender-based violence
SRH: Sexual and reproductive health
SSA: Sub-Saharan Africa
STI: Sexually transmitted infection
UNFPA: United Nations Population Fund
UNHCR: United Nations High Commissioner for Refugees
