# Supplementary material for: Health system resilience during COVID-19 understanding SRH service adaptation in North Kivu
Source: Reprod Health. 2022 Jun 6;19:135. doi: 10.1186/s12978-022-01443-5 (PMC9169445; doi:10.1186/s12978-022-01443-5)
Supplement: Supplementary file 1 — Additional file 1. Annex 1: Interview Guides in French and English. Annex 2: Coding Framework. [file 12978_2022_1443_MOESM1_ESM.docx]

**Annex 1: Interview Guides in French and English**

***Guide d’entretien***

# *Notez les détails des participants avant l’entretien :*

| 1. ID de l'entretien |  |
| --- | --- |
| 1. Date de l'entretien |  |
| 1. Genre | Homme  Femme  |
| 1. Titre de l'interviewé |  |
| 1. Institution/Organisation/ Département |  |
| - Niveau nationale, provincial ou zonal |  |

*Confirmez que la fiche d'information a été lue et comprise, posez d'autres questions - vérifiez que le formulaire de consentement a été signé - vérifiez / demandez le consentement pour l'enregistrement de l'entretien.*

*N'oubliez pas d'adapter et de personnaliser les questions en fonction des antécédents de l'informateur clé, de son expérience et de ses connaissances potentielles.*

1. Pouvez-vous me dire quel est votre **rôle professionnel actuel** ? Quel est votre rôle / vos tâches **par rapport à la prestation de services de SSR en** particulier ? Depuis combien de temps travaillez-vous dans ce poste ?

[Rechercher le type de rôle / d'affiliation - voir les options ci-dessous :

- responsable des politiques au ministère de la Santé et / ou dans d'autres institutions compétentes

- directeur de la santé au niveau provincial ou zonale

- personnel d'une ONG nationale et internationale

- personnel de l'agence des Nations Unies

- bailleur de fonds travaillant au niveau des pays

- représentant d'un prestataire de soins de santé privé et privé à but non lucratif / confessionnel

- représentant d'une organisation de la société civile ou d'une organisation communautaire

- autre]

- De votre point de vue, pourriez-vous décrire *brièvement* la **réponse du système de santé à l'épidémie de COVID-19** ?

- Nous entendons par ça, quels ont été les principaux changements dans le fonctionnement du système de santé pendant l'épidémie de COVID-19 ? Y a-t-il eu des changements dans les services offerts à la population, dans la manière dont les patients accèdent aux services et comment les services sont financés ? Y a-t-il eu des changements dans le déploiement et les tâches des agents de santé ? Qu'est-ce qui a changé en termes de disponibilité et d'approvisionnement en médicaments essentiels ?

*[Cela est une question pour comprendre les adaptations de la SSR dans le contexte général, pas besoin de trop de détails]*

- Pour cette étude, nous souhaitons nous concentrer en particulier sur les services de SSR et la fourniture du DMU. Quels ont été les **changements dans les services de SSR** depuis le début de l'épidémie de COVID-19 ?

- **Qu'est**-**ce qui** a changé en termes de politiques et de protocoles en place ? [*Demander également les modalités de financement et les modifications apportées à celles-ci]*

- **Quand** ces changements ont-ils été apportés ?

- Est-ce que les changements ont été révisés plusieurs fois dans le temps ? Si oui, veuillez décrire quand / comment.

*L'utilisation d'une chronologie (timeline) peut aider à mieux se souvenir et à obtenir plus de détails. Si l'interview est sur Zoom / Skype et que la connexion est bonne, vous pouvez partager l'écran et utiliser Google Jamboards. Par téléphone / WhatsApp, un document / chronologie peut être partagé à l'avance pour que le répondant puisse commenter. Sinon, vous pouvez également utiliser une chronologie pour prendre des notes de la réponse, si cela vous aide.*

1. Quel a été le **processus** qui a conduit au(x) changement(s) des politiques et protocoles ?

- Est-ce que des **réunions conjointes** ont été organisées ? Est-ce que ces réunions étaient gérées/organisées par des comités de coordination existants ou des nouveaux ont été créés ?

- **Qui** a participé à ces réunions ? [Si *possible, renseignez-vous sur leurs « caractéristiques », y compris le sexe et le titre / diplôme / formation des participants]*. Est-ce que la composition des participants est différente de celle des réunions précédentes a la COVID-19, menées pour réviser les protocoles de SSR ? Si c'est le cas, comment la composition a changée ?

- Quelles **preuves / évidence** (par exemple, données locales ou directives internationales) ou **informations** (par exemple, provenant de patients et de communautés) étaient disponibles au cours de ces réunions ? Comment été utilisé cette information lors du processus décisionnel ?

1. Quelle était la **justification et les raisons** qui ont été discutées pour soutenir et justifier les adaptations (ou le manque d'adaptation) ?

- Quel est votre point de vue sur ce raisonnement ?

- Auriez-vous fait les choses différemment ? Si oui, à quel égard ?

1. Comment ont été les adaptations aux protocoles SSR mises en œuvre dans la pratique ?

- Quels sont les principaux goulots d'étranglement et défis ?

- Quelle est l'opinion des prestataires / agents de santé au sujet de la mise en œuvre ?

- Comment les perspectives des agents des sante ont influencée la mise en œuvre ?

1. Selon vous, quelles sont les conséquences de l’adaptations des services/protocoles SSR en termes de l’état de santé des femmes et filles ?

- Y a-t-il eu des changements en termes d'accès aux services de SSR, y compris l'accès financier ?

- Est-ce que certains groupes sociaux, régionaux ou autres (ethniques, handicapés, déplacés, vulnérables) ont été inclus ou exclus ?

- Comment les femmes et filles ont fait face et géré ces changements ? Quels comportements ont-elles adoptés pour s'adapter aux changements ? [Par exemple, éviter les centres de santé et chercher des soins ailleurs, ou d’avantage des grossesses parce que la contraception n'est pas disponible, etc.].

1. Quels défis persiste pour les femmes dans le domaine de SSR à cause de COVID-19 ?
   - 1. Comment le système sanitaire et différents acteurs ont répondu aux ces défis ?
     2. Qu’est-ce qui reste à faire ?

1. Comment pensez-vous que le contexte et l'histoire récente du Nord-Kivu ont influencé la réponse de la SSR à la pandémie de COVID-19 ? Par exemple,

- Y-a-t ’il des leçons tirées de l'épidémie de EVD ?

- Comment la fragilité contextuelle / crise prolongée / (conflit actif dans certains cas) a-t-elle influencé la réponse SSR au COVID-19 ? [*Demander d’avantage des explications sur le rôle des acteurs / donateurs humanitaires et externes, sur les points de vue locaux et internationaux sur la SSR/VBG dans un contexte de crise et en particulier dans les Kivus, etc.]*

- Comment les valeurs / croyances des différents acteurs ont-elles façonné les points de vue sur la SSR et les adaptations de protocole ? [*Enquête sur le rôle des organisations catholiques, des ONG internationales et de la société civile, le cas échéant*].

1. Avez-vous des conclusions ou des recommandations à formuler ?

- Quels sont, selon vous, les principaux défis ou opportunités rencontrés pour garantir un accès continu aux services de santé sexuelle et reproductive, et fournir des recommandations potentielles ?

***FIN DE L'ENTRETIEN***

*Remerciez les participants pour leur temps.*

*Partager des informations et des contacts pour un engagement à plus long terme et la diffusion des résultats.*

*Note details of participants before interview:*

| 1. Interviewee ID |  |
| --- | --- |
| 1. Date of Interview |  |
| 1. Gender | Male □ Female □ |
| 1. Title of interviewee |  |
| 1. Institution / Organization / Department |  |
| 1. Central, Province or Zone |  |

*Confirm that information sheet has been read and understood, ask for any further questions – check that consent form has been signed – check/ask for consent for recording the interview.*

*Remember to adapt and tailor questions according to background of the key informant, their experience and potential knowledge.*

1. Could you please tell me what is your **current professional role**? What is your role/tasks **in relation to SRH service provision** specifically? How long have you been working in this position?

[Probe for type of role/affiliation – see options below:

- policy-maker at the Ministry of Health and/or other relevant institutions
- health manager at provincial or zonal level
- staff at national and international NGO
- staff at UN agency
- donor working at country level
- representative of a private and private non-for-profit healthcare provider
- representative of a civil society organisation or a community based organisation
- other]

1. From your perspective, could you *briefly* describe the **health system response to the COVID-19 epidemic**?

- By this we mean, what were the main changes to how the health systems work during the COVID-19 outbreak? Were there changes in the services available to the population, in how patients access services and how they pay for them (or not)? Were there changes in the deployment and task of health workers? What changed in terms of availability of essential drugs and supply?

*[this is a question to set the SRH adaptations against the general context, no need for too many details]*

1. For this study, we want to focus in particular on SRH services and the provision of the MISP. What were the **changes in SRH services** since the start of the COVID-19 epidemic?

- **What** has changed in terms of policies and protocols in place? [*also probe for funding arrangements and changes to those*]
- **When** were these changes made?
- Were these changes reviewed at different points in time? If yes, please describe when/how.

*Using a timeline might help better recalling and eliciting more details. If the interview is one on Zoom/Skype and the connection good, you can share the screen and use Google Jamboards. Via phone/WhatsApp, a document/timeline can be shared in advance for the respondent to comment on. Otherwise, you can also use a timeline to take notes of the response, if that helps.*

1. What was the **process** that led to the change(s) in policies and protocols?

- Were **joint meetings** organized? Was that done via existing coordination committees or new/*ad hoc* ones were created?
- **Who** participated to these meetings? [*if possible, ask about their ‘characteristics’, including gender and title/degree/training*]. Is this different from previous changes to SRH protocols? If so, how?
- What were their respective positions/views on the changes needed or not? Was there general agreement or some differing/conflicting perspectives? [*probe for the reasons of their views/actors’ agendas, including potential ‘winners’/’losers’ from changes, for example in terms of funding, rent seeking/patronage networks*]
- **Why** did some views prevail over others? How did different groups influence the decision-making process? [*probe for issues around power and influence here, including official funding or unofficial payments/corruption*]
- What **evidence** (e.g., local data or international guidance) or **information** (for example, from patients and communities) was available during those meetings? How was it used during the decision-making process?

1. What was the **rationale and reasons** that were discussed to support and justify the adaptations (or lack of adaptation)?

- What is your perspective and view on this rationale?
- Would have you done things differently? If so, in what respect?

1. How were the SRH adaptations implemented in practice?

- Which are the main bottlenecks and challenges?
- What are the views of health providers/health workers on them?
- How did these views influence implementation?

1. What do you think are the consequences of the implementation of the SRH adaptations in terms of health outcomes for women and girls?

- Were there any changes in terms of access to SRH services, including financial access?
- Are particular social, regional or specific groups (ethnic, disabled, displaced, vulnerable) included or excluded?
- How women and girls have coped? What measures/behaviors are they taking to adapt to the changes? [For example, avoiding health facilities or seeking care elsewhere, or having more pregnancies because contraception is not available, increased access to safe abortion self-care pilot].

1. How do you think the context and recent history of North Kivu influenced the SRH response to the COVID-19 pandemic? For example,

- Were there any lessons learned from the EVD epidemic?
- How did the contextual fragility/protracted crisis/ (active conflict in some cases) influence the SRH response to COVID-19? [*probe for role of humanitarian and external actors/donors,* *local and international views of SRH/GBV in crisis context and specifically in the Kivus, etc.]*
- How did the values/beliefs of different actor’s shape views around SRH and protocol adaptations? [*probe for role of Catholic organizations, INGOs and civil society, if relevant*].

1. Do you have any concluding thoughts or recommendations to make?

- What do you think are the main challenges or opportunities being faced with regards to ensuring continued access to sexual and reproductive health services, and provide potential recommendations?

***END OF INTERVIEW***

*Thank participant for their time.*

*Share information and contacts for longer-term engagement and dissemination of results.*

**Annex 2: Coding Framework**

| **Main theme** | **Sub-theme** | **Comments** |
| --- | --- | --- |
| Changes in access to/utilization of SRH services | Changes in utilization of SRH services *due to COVID-19* | Include any information, including anecdotal on changes in utilization rates that might be related to COVID-19 |
|  | Changes on access to/utilization of SRH services *due to other causes* | Include any information, including anecdotal on changes in utilization rates that might be related to other causes. |
|  | Health seeking behavior | Include information on any changes to health seeking behavior of patients/users that might influence utilization rate |
| Impact of COVID-19 on SRH needs | -- | Include any information, including anecdotal on changes in SRH needs for women and girls that might be related to COVID-19 |
| COVID-19 guidelines and protocols | COVID-19 guidelines and protocols (SRH and beyond) | Include any reference to protocols/guidelines as designed/envisaged and description of measures included in those and the targets of those measures |
|  | Actors involved in designing COVID-19 guidelines | Include actors at national and provincial level involved in designing COVID-19 protocols. |
|  | (planning) processes for designing COVID-19 guidelines | Include description of processes and coordination forums/meetings/groups where COVID-19 protocols were designed/agreed on. Also include role evidence/information on utilization and needs in shaping guidelines. |
| Implementation and adaptations to SRH services | Changes in SRH service delivery at facility level | Include both changes that are due to public health/IPC measures (social distancing, hand washing, triage) and those which are a response of facilities to those changes (e.g., extending opening times, scheduling appointments, etc.). |
|  | Changes in SRH service delivery at community level | As above |
|  | Differences between actors/facilities in terms of SRH adaptation and implementation |  |
|  | Actors involved in implementing changes | Who decided on those changes? How? Who is responsible of decision making? How were changes communicated (unless decided at delivery level)? |
|  | (planning) processes for defining SRH adaptations | Include description of processes and coordination forums/meetings/groups where SRH-specific adaptation and implementation issues were discussed (vs. focus on design in code above). Also include role evidence/information on utilization and needs in shaping adaptations decisions. |
| Role of different elements in implementation of SRH adaptation and service delivery | Funding / lack of funding | Can be challenges/ bottlenecks or elements supporting adaptation (and resilience) |
|  | Availability of materials / inputs |  |
|  | Level of prioritization of SRH during COVID-19 response |  |
|  | Coordination / fragmentation |  |
|  | Learning processes |  |
|  | Technical knowledge and evidence |  |
|  | EVD epidemic |  |
|  | Beliefs and views of communities |  |
|  | Health workers (including skills/capacity, motivation and beliefs/views) |  |
|  | Consequences of lockdown and other public health measures |  |
|  | Other elements |  |
| Assessment of response | Effectiveness of SRH changes to address needs | Considerations/perceptions/views of the respondent on how SRH service needs are met through those adaptations, or conversely on gaps in SRH service delivery |
